# Data mining methods in the prediction of Dementia: A real-data comparison of the accuracy, sensitivity and specificity of linear discriminant analysis, logistic regression, neural networks, support vector machines, classification trees and random forests

**DOI:** 10.1186/1756-0500-4-299

**Published:** 2011-08-17

**Authors:** João Maroco, Dina Silva, Ana Rodrigues, Manuela Guerreiro, Isabel Santana, Alexandre de Mendonça

**Affiliations:** 1 Unidade de Investigação em Psicologia e Saúde & Departamento de Estatística, ISPA - Instituto Universitário, Rua Jardim do Tabaco 44, 1149-041 Lisboa. Portugal; 2 Instituto de Medicina Molecular, Faculdade de Medicina, Universidade de Lisboa. Av. Professor Egas Moniz, 1649-028 Lisboa, Portugal; 3 Departamento de Neurologia, Hospitais da Universidade de Coimbra, Praceta Prof. Mota Pinto, 3000-075 Coimbra, Portugal

## Abstract

**Background:**

Dementia and cognitive impairment associated with aging are a major medical and social concern. Neuropsychological testing is a key element in the diagnostic procedures of Mild Cognitive Impairment (MCI), but has presently a limited value in the prediction of progression to dementia. We advance the hypothesis that newer statistical classification methods derived from data mining and machine learning methods like Neural Networks, Support Vector Machines and Random Forests can improve accuracy, sensitivity and specificity of predictions obtained from neuropsychological testing. Seven non parametric classifiers derived from data mining methods (Multilayer Perceptrons Neural Networks, Radial Basis Function Neural Networks, Support Vector Machines, CART, CHAID and QUEST Classification Trees and Random Forests) were compared to three traditional classifiers (Linear Discriminant Analysis, Quadratic Discriminant Analysis and Logistic Regression) in terms of overall classification accuracy, specificity, sensitivity, Area under the ROC curve and Press'Q. Model predictors were 10 neuropsychological tests currently used in the diagnosis of dementia. Statistical distributions of classification parameters obtained from a 5-fold cross-validation were compared using the Friedman's nonparametric test.

**Results:**

Press' Q test showed that all classifiers performed better than chance alone (p < 0.05). Support Vector Machines showed the larger overall classification accuracy (Median (Me) = 0.76) an area under the ROC (Me = 0.90). However this method showed high specificity (Me = 1.0) but low sensitivity (Me = 0.3). Random Forest ranked second in overall accuracy (Me = 0.73) with high area under the ROC (Me = 0.73) specificity (Me = 0.73) and sensitivity (Me = 0.64). Linear Discriminant Analysis also showed acceptable overall accuracy (Me = 0.66), with acceptable area under the ROC (Me = 0.72) specificity (Me = 0.66) and sensitivity (Me = 0.64). The remaining classifiers showed overall classification accuracy above a median value of 0.63, but for most sensitivity was around or even lower than a median value of 0.5.

**Conclusions:**

When taking into account sensitivity, specificity and overall classification accuracy Random Forests and Linear Discriminant analysis rank first among all the classifiers tested in prediction of dementia using several neuropsychological tests. These methods may be used to improve accuracy, sensitivity and specificity of Dementia predictions from neuropsychological testing.

## Background

It is estimated that about 25 million people suffer from dementia nowadays and, as a consequence of the population aging, the number of people affected is expected to double every 20 years [1]. The presence of cognitive complaints is very common in aged people and may be the first sign of an on-going dementing disorder like Alzheimer's disease. It is possible to identify people with cognitive complaints who are at risk for the progression to dementia, that is to say, who have Mild Cognitive Impairment (MCI) [2,3]. Since the establishment of MCI requires the demonstration of cognitive decline greater than expected for an individual's age and education level, neuropsychological testing is a key element in the diagnostic procedures [4].

Recently, it has become possible to identify the traces, or biomarkers, of Alzheimer's disease in patients with MCI, by the use of Magnetic Resonance Imaging (MRI) volumetric studies, neurochemical analysis of the cerebrospinal fluid, and Positron Emission Tomography (PET) scan [5]. These studies, however, are expensive, technically challenging, some invasive, and not widely available. Longitudinal studies assessing the predictive value of neuropsychological tests in progression of MCI patients to dementia have shown an area under the receiver operating characteristic curve of 61-94% (being higher for tests assessing verbal episodic memory) but with lower accuracy and sensitivity values [6-11]. It would be important to improve the value of neuropsychological tests to predict the progression of MCI patients to dementia. This can be achieved at a clinical level by increasing the number of patients with longer clinical follow-ups. Predictive power of these tests may be also enhanced through innovating statistical classification and data mining techniques. Traditional statistical classification methods (e.g., Fisher's Linear Discriminant Analysis (LDA) and Logistic Regression (LR)) have been extensively used in medical classification problems for which the criterion variable is dichotomous [12-18]. More recently, research has been steadily building on the accuracy and efficiency of data mining, with classifiers like Neural Networks (NN), Support Vector Machines (SVM), Classification Trees (CT) and Random Forests (RF) used for medical prediction and classification tasks [13,14,19-27]. Research on the comparative accuracy of traditional classifiers (LDA and LR) vs. new, computer intensive data mining methods which require large computing power, innovative iterative algorithms and user intervention, has been growing steadily. Several authors propose that data mining classifiers have higher accuracy and lower error rates than the traditional classification methods [22,25,28,29]. However, this superiority is not apparent with all data sets, especially with real data [12,13,30-32]. Results regarding the superiority of classification accuracy of newer classification methods as compared to traditional, less computer demanding methods, as well as the stability of the findings are still controversial [31,33-35]. Most comparisons between methods are based only on total classification accuracy and/or error rates; they involve human intervention for training and optimization of the data mining classifiers vs. out-of-the-box results for the traditional classifiers. Furthermore, in medical contexts, sensitivity (the ability to predict the condition when the condition is present), specificity (the ability to predict the absence of the condition when the condition is not present) as well as the classifier discriminant power (as estimated from the area under the Receiver Operating Characteristic (ROC) curve) are key features that must be considered when comparing classifiers and diagnostic methods.

In this paper we evaluated the sensitivity, specificity, overall classification accuracy, area under the ROC and Press' Q of data mining classifiers like Neural Networks (Multilayer Perceptrons and Radial Basis Networks), Support Vector Machines, Classification Trees and Random Forests as compared to the traditional Linear, Quadratic Discriminant Analysis and Logistic Regression in the prediction of the evolution into dementia of 400 elderly people with Mild Cognitive Impairment.

## Methods

### Classifiers

#### Discriminant Analysis

The oldest classifier still in use was devised almost 100 years ago by Sir R. Fisher [36]. Fisher's Linear Discriminant Analysis (LDA) builds *j *= min(*k*-1*,p*) discriminant functions that estimate discriminant scores (*D_ji_*) for each of *i = *1,...,*n* subjects classified into *k *groups, from *p *linearly independent predictor variables (**X**) as

$$D_{ji}=w_{i1}X_{1i}+w_{i2}X_{2i}+\ldots +w_{ip}X_{pi}$$

[i=1,…,nandj=1,…,min(k-1,p)]

Discriminant weights (*w_ij_*) are estimated by ordinary least squares so that the ratio of the variance within the *k *groups to the variance between the *k *groups is minimal. Classification functions of the type

$$C_{ji}=c_{jo}+c_{j1}X_{1i}+c_{j2}X_{2i}+\ldots +c_{jp}X_{pi}$$

for each of the *j *= 1,...,*k *groups can therefore be constructed from the discriminant scores. The coefficients of the classification function for the *j *th group are estimated from the within sum of squares matrixes (**W**) of the discriminant scores for each group and from the vector of the *p *discriminant predictors means in each of the classifying groups (**M**) as **C***_j _*= **W**^-1^**M **with cjo=logp−12CjMj. Quadratic Discriminant Analysis (QDA) uses the same within vs. between sum of square minimization optimization but on a quadratic discriminant function of the form:

$$\begin{array}{lll} D_{i}=\overset{P}{\underset{p=1}{\sum }}w_{\overset{{}^{\circ}}{\iota }p}X_{p}+\overset{P}{\underset{p=1}{\sum }}q_{\overset{{}^{\circ}}{\iota }p}X_{p}^{2}+\overset{P-1}{\underset{p=1}{\sum }}r_{\overset{{}^{\circ}}{\iota }p}X_{p}X_{p+1} & \; & \text{(1)}\\ \left[i=1,\ldots ,\;\min\left(k-1,p\right)\right] & \; & \text{(2)}\\ & \; & \text{(3)} \end{array}$$

With classification functions

$$\begin{array}{c} c_{j}=c_{0j}+\overset{P}{\underset{p=1}{\sum }}c_{\overset{{}^{\circ}}{\iota }p}X_{p}+\overset{P}{\underset{p=1}{\sum }}o_{\overset{{}^{\circ}}{\iota }p}X_{p}^{2}+\overset{P-1}{\underset{p=1}{\sum }}m_{\overset{{}^{\circ}}{\iota }p}X_{p}X_{p+1}\\ \left[j=1,\ldots ,k\right] \end{array}$$

Both on LDA and QDA, a subject is then classified into the group for which its classification function score is higher [for a detailed description of LDA and QDA see [37]].

#### Logistic Regression

Binomial Logistic regression (LR) models the probability of occurrence of one (success) of the two classes of a dichotomous criterion. A linear combination of predictors is used to fit a Logit transformation of the probability of success for each subject (π*_i_*) as

$$Ln[{\hat{\pi }}_{i}/(1-{\hat{\pi }}_{i})]=\beta_{o}+\beta_{1}X_{1i}+\ldots +\beta_{p}X_{pi}$$

Regression coefficients are fitted by maximum likelihood estimation, and by solving the Logit in order to π*_i _*the probability of success for each subject is estimated as

$${\hat{\pi }}_{i}=\frac{e^{\beta_{0}+\beta_{1}X_{1i}+\ldots +\beta_{p}X_{pi}}}{1+e^{\beta_{0}+\beta_{1}X_{1i}+...+\beta_{p}X_{pi}}}$$

If the estimated probability is greater than 0.5 (or other user pre-defined threshold value), the subject is classified into the success group; otherwise, it is classified into the failure group [for a detailed description see [38]].

#### Neural Networks

**Figure 1:**
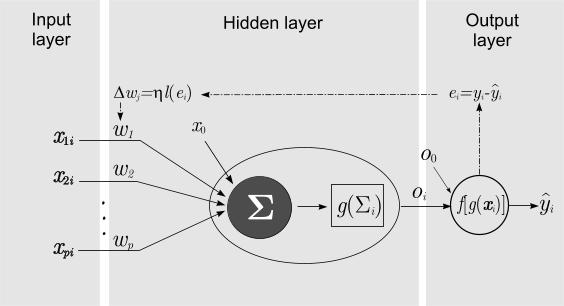
**Pictorial representation of a neural network (multilayer perceptron) with input layer (dendrites), hidden layer (nucleus) and output layer (axon) (see text for a description of the neural networks components)**.

Neural Networks (NN) methods have been used extensively in classification problems and this is one of the most active research and application areas in the Neural Networks field [39]. Inspired from the biological neuron cells, a NN is a multi-stage, multi-unit classifier, with input, hidden or processing, and output layers as illustrated by Figure 1.

For a polytomous criterion *y_k _*with *k *classes, the NN can be described by general the model

$$\begin{array}{ll} {\hat{y}}_{k} & =f_{k}(\text{x},\text{w},\text{o},{\text{x}}_{0},{\text{o}}_{0k},\theta )=\\ & =f\left(\sum_{j=1}^{h}o_{kj}\cdot g\left(\sum_{i=1}^{p}w_{ji}{\text{x}}_{i}+x_{0j}\right)+o_{0k}\right) \end{array}$$

Where **x **is the vector of *p *predictors, **w **is the vector of input weights, **o **is the vector of hidden weights for the hidden layer, **x**_0 _and **o**_0k _are bias (memory) constants. The functions *g*(.) and *f*(.) are processing activation functions for the hidden layer and output layer respectively. Activation functions are one of the general linear, logistic, exponential or gaussian function families. Several topologies of Neural Networks (NN) can be used in binary classification problems. Two of the most used NN are the Multilayer Perceptron (MLP) and the Radial Basis Function (RBF). The main differences between these two NN reside in the activation functions of the hidden layer: For the MLP the activation function belongs, generally, to a linear

$$f_{j}\left(x\right)=\sum_{i=1}^{p}w_{ij}x_{i}$$

or logistic activation function family:

$$f\left(x\right)=\frac{1}{1+\exp\left(-x\right)}$$

For the RBF function the activation function belongs to the Gaussian family:

$$f_{j}\left(x\right)=\exp\left[-\frac{1}{2}{\left(x-\mu_{j}\right)}^{\prime }\Sigma_{j}^{-1}\left(x-\mu_{j}\right)\right]$$

A NN is generally trained in a set of iterations (epochs) for a subset of the data (train set) and tested for the remained subset (test set). The vector of sinaptic weights (**w**) of the NN is upgraded in each iteration in way to maximize the correct classification rate and or minimize a function of the classification errors; either a function of the sum of squares of the errors for a continuous criterion

$$SSE=\frac{1}{2}\overset{n}{\underset{i=1}{\sum }}{\left(y_{i}-{ŷ}_{i}\right)}^{2}$$

or the Cross-entropy error function for a binary criterion:

$$CEE=-\overset{n}{\underset{i=1}{\sum }}\left[y_{i}1\;Ln\left(\frac{{ŷ}_{i}}{y_{i}}\right)+\left(1-Y_{i}\right)Ln\frac{\left(1-{ŷ}_{i}\right)}{\left(1-y_{i}\right)}\right]$$

[for a detailed description of NN see [40]].

#### Support Vector Machines

Support Vector Machines (SVM) are machine-learning derived classifiers which map a vector of predictors into a higher dimensional plane through either linear and non-linear kernel functions [41]. In a binary classification problem, the two groups, say {-1} and {+1}, are separated in a higher-dimension hyperplane accordingly to a structural risk minimization principle. The objective is to find a linear separating hyperplane

$$w^{\prime }\phi \left(x\right)+b=0$$

constructed from a vector **x **of predictors mapped into a higher dimension feature space by a nonlinear feature function *ϕ*, a vector **w **of weights and a bias offset *b*, that classifies all the observation *y_i _*in one of the two groups {-1; +1} [41]. The classification function is then

$$f\left(x\right)=Sign\left(w^{\prime }\phi \left(x\right)+b\right)$$

**Figure 2:**
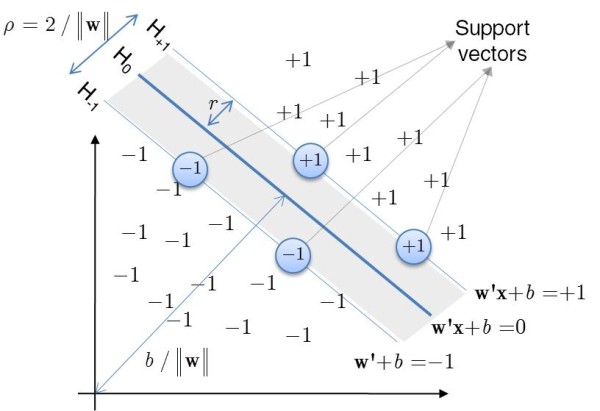
**Schematic representation of the optimum hyperplane (H0) by a Support Vector Machine**. Diagonal lines represent the classification function for objects {-1} and {+1}. Objects inside the circles are the so-called support vectors verifying **w**'**x **+ *b *= -1 or **w**'**x **+ *b *= + 1 respectively.

Since, in a binary classification problem, there are infinite separation hyperplanes, the goal is to find the optimum linear plane which separates best the two groups. To find the optimum plane furthest from both {-1} and {+1} groups, one strategy is to maximize the distance or margin of separation from the supporting planes, respectively **w**'*ϕ*(**x**) + *b *≥ +1 for the {+1} group and **w**'*ϕ*(**x**) + *b *≤ -1 for the {-1} group. These support planes are pushed apart until they bum into a small number of observations or training patterns that respect the above constrains and thus are called support vectors. Figure 2 illustrates this concept. The classification goal can be achieved by maximizing the distance or margin of separation *r *between the two planes **w**'*ϕ*(**x**) + *b *= +1 and **w'x **+ *b *= -1 given by *r *= 2/|| **w **||. This is equivalent to minimizing the cost function

$$C\left(w\right)=\frac{{∥w∥}^{2}}{2}+c\overset{n}{\underset{i=1}{\sum }}\xi_{i}=\frac{1}{2}w^{\prime }w+c\overset{n}{\underset{i=1}{\sum }}\xi_{i}$$

Subjected to the linear inequality constrains

$$y_{i}\left(w^{\prime }\phi \left(x_{i}\right)+b\right)\geq 1-\xi_{i}\;\text{and}\;\xi_{i}\geq 0$$

where *c *> 0 is penalty parameter that balances classification errors vs. the complexity of the model, which is controlled by the margin of separation, and *ξ_i_*, is the so called slack-variable. This variable is the penalty of a misclassified observation that controls how far on the wrong side of the hyperplane a point can lie when the training data cannot be classified without error, that is when the objects are not linearly separable and a soft separating non-linear margin is required [41,42]. Because the feature space can be infinite, the nonlinear mapping by the feature function *ϕ *is computed through special nonlinear semi-positive definite K functions called kernels (Ivanciuc, 2007).

Thus, the above minimization is generally solved through a dual formulation problem [see e.g. [41,43]]:

$$\min\frac{1}{2}\overset{n}{\underset{i,j=1}{\sum }}y_{i}y_{j}\alpha_{i}\alpha_{j}\text{K}\left(x_{i},x_{j}\right)-\overset{n}{\underset{i=1}{\sum }}\alpha_{i}$$

subjected to the linear constrains

$$\overset{n}{\underset{\overset{{}^{\circ}}{\iota }=1}{\sum }}y_{i}\alpha_{i}=0\;\text{and}\;0\leq \alpha_{i}\leq C$$

Where *α_i_*(*i *= 1,...,*n*) are nonnegative Lagrange multipliers and K(.) is a kernel unction. In classification problems (c-SVM) the usual kernel functions are the linear kernel K(**x***_i_*, **x***_j _*) = **x***_i _***'x***_j _*or the Gaussian K(**x***_i_*, **x***_j_*) = exp(-*γ *||**x***_i _*- **x***_j_*||^2^) where γ is the kernel parameter. The use of kernel functions has the advantage of operating in the original input variables where the solution of the classification problem is a weighted sum of kernels evaluated at the support vectors [for a complete description of SVM see [28,41,43].

#### Classification Trees

Classification Trees (CT) are non-parametric classifiers that construct hierarchical decision trees by splitting data among classes of the criterion at a given step (node) accordingly to an "if-then" rule applied to a set of predictors, into two child nodes repeatedly, from a root node that contains the whole sample. Thus, CT can select the predictors and its interactions that are most important in determining an outcome for a criterion variable. The development of a CT is supported on three major elements: (1) choosing a sampling-splitting rule that defines the tree branch which connect the classification nodes; (2) the evaluation of classification produced by the splitting rule at each node and (3) the criteria used for choosing an optimal or final tree for classification proposes. Accordingly to the features of these major elements, the most usual CT can be classified into: Classification and Regression Tree (CART) [44], Chi-squared Automatic Interaction Detector (CHAID) [45] and Quick Unbiased Efficient Statistical Tree (QUEST) [46]. The following descriptions are based on these algorithms and its references. In CART trees, the predictors are split in a way that minimizes the impurity of node produced at each *t *branch of the tree until all data points are classified into *C *mutually exclusive classes. The impurity measure of choice in CART is the Gini impurity index defined as

$$\begin{array}{ll} I_{G}(t) & =1-\sum_{c=1}^{C}P{\left(c|t\right)}^{2}=\\ & =\sum_{c=1}^{C}\sum_{c\neq d=1}^{C}P(c|t)P(j|t) \end{array}$$

where *P *(*c *| *t*) is the conditional probability of a class *c *given the node *t*. This probability is estimated as

$$\begin{array}{c} P\left(c|t\right)=\frac{P\left(c,t\right)}{P\left(t\right)}\\ \text{with}\;P\left(c,t\right)=\frac{\pi \left(c\right)n_{c}\left(t\right)}{n_{c}}\;\text{and}\;P\left(t\right)=\overset{C}{\underset{c=1}{\sum }}P\left(c,t\right) \end{array}$$

where π(*c*) is the probability of observing the group *c *and *n*_c_(*t*) is the number of elements in group *c *at a given node *t*. The tree is grown until no further predictors can be used or the impurity of each group at a final branch of the tree cannot be reduced further. Non significant predictors (branches) can be pruned from the final tree and removed from the analysis.

In CHAID trees, the homogeneity of the groups generated by the tree is evaluated by a Bonferroni corrected *p-value *obtained from the chi-square statistic applied to two-way classification tables with *C *classes and *K *splits for each tree node:

$$X^{2}=\overset{C}{\underset{c=1}{\sum }}\overset{K}{\underset{k=1}{\sum }}\frac{{\left(n_{ck}-{\hat{n}}_{ck}\right)}^{2}}{{\hat{n}}_{ck}}\sim \chi_{\left(C-1\right)\left(K-1\right)}^{2}$$

where *n_ck _*stands for the observed frequencies of cell *ck *and ${\hat{n}}_{ck}$ stands for the expected frequencies under the null hypothesis of two-way homogeneity.

In QUEST, the homogeneity of groups at each branch of the tree is evaluated with the ratio of the within group variance and between group variances for continuous predictors which define the *F *statistic:

$$F_{X}=\frac{\overset{C}{\underset{c=1}{\sum }}n_{c}\left(t\right)\frac{{\left({\bar{x}}_{c}\left(t\right)-\bar{x}\left(t\right)\right)}^{2}}{\left(C-1\right)}}{\overset{n}{\underset{i=1}{\sum }}\frac{\left(x_{i}-{\bar{x}}_{c}\left(t\right)\right)}{\left(n\left(t\right)-C\right)}}\sim F\left(C-1;n\left(t\right)-C\right)$$

where ${\bar{x}}_{c}\left(t\right)$ is the average of predictor *X *in the *c *group at node *t *and $\bar{x}\left(t\right)$ is the average of predictor *X *at node *t *for all groups. For categorical predictors, a chi-square like statistic similar to the one defined for a CHAID is used.

#### Random Forests

Random Forests (RF) were proposed by Leo Breinman [47]. This "ensemble learning" classification method construct a series of CART using random bootstrap samples of the original data sample. Each of these trees is built from further random sub-set of the total predictors who maximize the classification criteria at each node. An estimate of the classification error-rate can be obtained using each of the CART to predict the data not in the bootstrap sample ("out-of-the bag") used to grow the tree, and then average the out-of-the bag predictions for the grown set of trees (forest). These out-of-the bag estimates of the error-rate can be quite accurate if enough trees have been grown [48]. Object classification is then performed from the majority of predictions given by the trees in the random forest. Although this classification strategy may lack a perceivable advantage over single CT, according to its creator (Leo Breiman), it has unexcelled accuracy among current algorithms, performing very well when compared to many classifiers including LDA, NN and SVM [for a detailed description of RF see [47]]. Furthermore, this method is quite user-friendly since it has only two parameters that the user needs to define: the number of random trees in the forest; and the number of predictor variables in the random subset of tree at each node. These parameters can be easily optimized although random forests are not very sensitive to their values [48].

### Case study application

#### Sample

Subjects were recruited as part of a cohort study of 921 elderly non-demented patients with cognitive complaints referred for neuropsychological evaluation at 3 institutions, the Laboratory of Language Studies, Santa Maria Hospital, and Memoclínica (a Memory Clinic), both in Lisbon, and the Neurology Department, University Hospital, Coimbra, from 1999 to 2007. Inclusion criteria consisted in the diagnosis of Mild Cognitive Impairment (according to the criteria of the European Consortium on Alzheimer's Disease, 2006); presence of at least one follow-up neuropsychological assessment or clinical re-evaluation. Patients with dementia [DSM-IV-TR [49]] or other disorders that may cause cognitive impairment, like stroke, brain tumour, significant head trauma, epilepsy, psychiatric disorders, uncontrolled medical illness (hypertension, metabolic, endocrine, toxic and infectious diseases); medical treatments interfering with cognitive function; and alcohol or illicit drug abuse were excluded from the study sample. At the follow-up, the subjects were classified as having: Mild Cognitive Impairment (according to the same criteria); or Dementia (DSM-IV-TR, 2000). The final sample was composed by 400 patients (see Table 1 for sample demographics) who gave voluntary consent to participate in this study. The local ethics committee approved the study.

**Table 1:** Sample demographics: The two groups in the criterion were "MCI" - Mild Cognitive impaired patients; and "Dementia" patients.

	MCI	Dementia	*p-value*
Group size (%)	275(69%)	125 (31%)	<0.001^‡^
Age (M ± SD)	67.8 ± 8.8	71.6 ± 8.4	<0.001^†^
Sex (♀/♂)	165/110	78/47	0.649^‡^
Schooling years (M ± SD)	8.1 ± 4.7	8.64 ± 4.9	0.469^†^
Time between assessments (year)(M ± SD)	2.3 ± 1.6	2.2 ± 1.4	0.517^†^

The class to predict was "Dementia". P-values for group comparison were obtained from Student's-t test (†) or χ^2 ^test (‡).

### Criterion and Predictors

**Figure 3:**
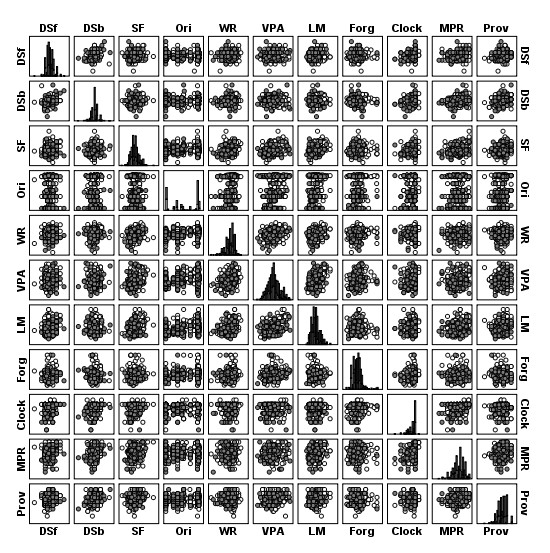
**Scatter biplots for MCI (white circles) and Dementia (black circles) patients in the 11 predictors and its histograms (DSf - Digit Span Forward; DSb - Digit Span Backward; SF - Verbal Semantic Fluency; Ori - Orientation; WR - Word Recall; VPA - Verbal Paired-associate Learning; LM - Logical Memory; Forg - Forgetting Index; Clock-Clock Drawing; MPR - Raven Progressive Matrices; Prov - Interpretation of Proverbs)**. See text for tests descriptions.

The criterion was a dichotomous variable with two groups: MCI and Dementia. Neuropsychological predictors were a subset of tests with criterion validity (p < 0.1) from the Battery of Lisbon for the Assessment of Dementia (BLAD) [50], which includes multiple neuropsychological tests representing key cognitive domains and was validated for the Portuguese population. The selected 10 neuropsychological tests assessed the following cognitive areas: verbal initiative (Verbal Semantic Fluency)[51]; verbal and non-verbal abstraction (Interpretation of Proverbs and the Raven Progressive Matrices)[52]; visuo-constructional abilities and executive functions (Clock Draw) [53]; immediate memory (Digit Span forward) [54]; working memory (Digit Span backward) [54]; learning and verbal memory (Word Recall, Verbal Paired-associate Learning and Logical Memory) [54] and orientation (adapted from the Mini-Mental State Examination (MMSE) Test) [50]. A Forgetting Index was also studied as a predictor variable. This Index is calculated based on the correct information evoked between the immediate and the delayed condition of the Logical Memory Test (Forgetting Index = [(LM delayed recall - LM immediate)/LM immediate)] × 100)[55] Figure 3 gives the scatter biplots for all pairs of predictors and their frequency histograms. None of the predictors showed a normal distribution judging from Kolmogorov-Smirnov with Lilliefors correction tests (p < 0.05), but criterion group variances were homogenous according to the Levene's test (p > 0.05). No multicollinearity problems were apparent (VIF<5) but several bivariate outliers were detected (see Figure 3).

### Data mining settings and classifiers evaluation

To prevent overfitting and artificial accuracy improvement due to the use of the same data for training and testing of classifiers, a 5-fold cross-validation strategy was followed to train and evaluate the 10 classifiers. The total sample was divided into 5 proportional sub-samples. In each of the 5 steps, 4/5 of the sample was used for training and 1/5 for testing. Test results for the 5 runs, gathered from the 5 test samples, were then considered for further comparisons. The performances (total accuracy, sensitivity, specificity, AUC and Press' Q) of the different classifiers where compared with Friedman's test followed by Dunn's post-hoc multiple comparisons of mean ranks for paired samples. Statistical significance was assumed for *p *< 0.05. To avoid biases from the data sets, equal a priori classification probabilities were used for Linear Discriminant Analysis, Quadratic Discriminant Analysis and Logistic Regression. Neural Networks, Support Vector Machines, Classification trees and Random forests used settings that are most frequently employed in practical data mining applications as follows. The Multilayer Perceptron was trained with 11 inputs (one for each predictor) in the input layer, 1 hidden layer with 4-7 neurons and a hyperbolic tangent activation function. The number of neurons in the hidden layer was iteratively adjusted by the software to minimize classification errors in the train data set. The activation function for the output layer was the Softmax with a cross-entropy error function. Synaptic weights were obtained from a 80%:20% train: test setup. The Radial Basis Function Neural Network had 11 inputs, one hidden layer with 2-8 neurons and a Softmax activation function. The activation function for the output layer was the identity function with a sum of squares error function. The Gaussian function was the kernel used in the SVM. Cost (*c*) and γ parameters were optimized by a linear grid search in the intervals [2^-3^; 2^15^] for *c *and [2^-15^; 2^3^] for γ, followed by cross-validation of each of the SVM obtained in the 5 train sets. The classification function was the sign of the optimum margin of separation. CHAID, CART and QUEST classification trees used α to split and α to merge of 0.05, with 10 intervals. Tree growth and pruning of CART were set with a minimum parent size of 5 and minimum child size of 1. Classification priors for both trees were fixed at 0.5:0.5. Random Forests were composed of 500 CART trees with 2-9 predictors per tree cross-validation optimization. The Predictive Analytic Software (PASW) Statistics (v. 18, SPSS Inc., Chicago, Il) was used for Discriminant Analysis, Logistic Regression, Neural Networks and Classification Trees. Support Vector Machines and Random Forests were performed with R (v. 2.8, CRAN) with the *e1071 *[56] and *randomForest *[48] packages, respectively.

## Results

Classification accuracy, sensitivity, specificity, area under the ROC and Press' Q statistic were evaluated in the 5 test sets resulting from the 5-fold cross validation strategy as described before. Data gathered is illustrated in box-plots for the different classifiers.

### Total Accuracy

**Figure 4:**
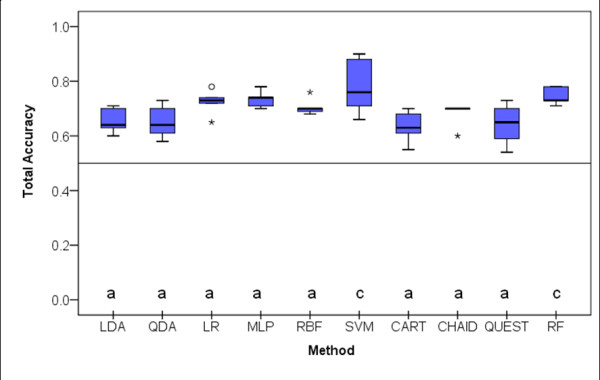
**Box-plot distributions of classification accuracy (number of correct classifications/total sample size) for the 5 test samples resulting from the 5-fold cross-validation procedure (see text for abbreviations) (X^2^_Fr_(9) = 22.211; *p *= 0.008)**. Different letters correspond to methods with statistically significant differences according to Dunn's mean rank post-hoc comparisons (p < 0.05). Circles represent outliers (observations greater than the 3^rd ^quartile plus 1.5 times the interquartile range or smaller than the 1^st ^quartile minus 1.5 times the interquartile range; stars represent extreme outliers, that correspond to observations greater than the 3^rd ^quartile plus 3 times the interquartile range or smaller than the 1^st ^quartile minus 3 times the interquartile range.

Figure 4 shows the box-plots of the total classification accuracy for the 10 classifiers studied. Judging from the Friedman's test on ranks, there were statistical significant differences between distributions of the total accuracy (X^2^_Fr_(9) = 22.211; *p *= 0.008). Post-hoc, multiple mean rank comparisons for paired samples revealed that SVM and RF had higher mean ranks than the other classifiers who did not differ significantly in mean rank accuracy (*p *> 0.05).

### Specificity

**Figure 5:**
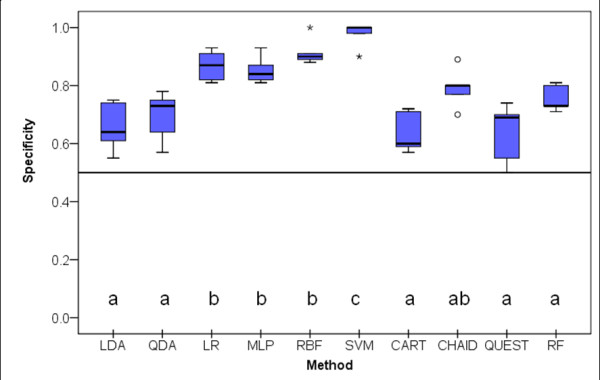
**Box-plot distributions of specificity (number of MCI predicted/number of MCI observed) for the 5 test samples resulting from the 5-fold cross-validation procedure (see text for abbreviations) (X^2^_Fr_(9)= 37.292; *p *< 0.001)**. Different letters indicate statistically significant differences between classifiers on Dunn's mean rank comparison procedure. Circles and stars represent outliers and extreme outliers respectively.

The distributions of the specificity (the proportion of subjects that did not convert to dementia and were correctly predicted) are shown in Figure 5. The differences in the specificity distributions were statistically significant (X^2^_Fr_(9)= 37.292; *p *< 0.001). SVM scored the highest in specificity followed by a second group composed by MLP, LR and RBF with significant differences from a third group composed by LDA, QDA, classification trees and RF.

### Sensitivity

**Figure 6:**
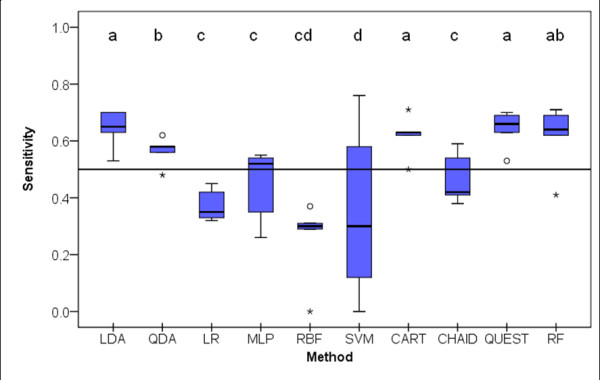
**Box-plot distributions of sensitivity (number of Dementia predicted/number of Dementia observed) (see text for abbreviations) (X^2^_Fr_(9)= 29.0; *p *= 0.001)**. Different letters indicate statistically significant differences between classifiers on a multiple mean rank comparison procedure. Circles and stars represent outliers and extreme outliers respectively.

Figure 6 illustrates the distributions of the sensitivity (proportion of subjects that were correctly predicted to convert into dementia) values obtained by the 10 classifiers in the 5 test samples. There were statistically significant differences in the distribution of the sensitivity values of the analyzed classifiers (X^2^Fr(9) = 29.0; *p *= 0.001). LDA, CART, QUEST and RF had the highest sensitivity values. It is worthwhile to mention that LR, MLP, RBF and CHAID had median sensitivity values close to or lower than 0.5, and that SVM was the classifier with the significantly lowest sensitivity.

### Area under the ROC

**Figure 7:**
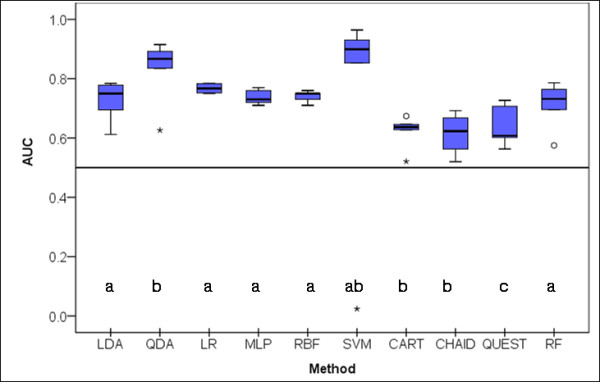
**Box-plot distributions of area under the Receiver Operating Characteristic curve (AUC) (see text for abbreviations) (X^2^_Fr_(9)= 23.745; *p *= 0.005)**. Different letters indicate statistically significant differences between classifiers on a multiple mean rank comparison procedure. Circles and stars represent outliers and extreme outliers respectively.

The distribution of the areas under the ROC (AUC) for the 10 classifiers in the 5 test samples is shown in Figure 7. There are statistically significant differences between the classifiers (X^2^_Fr_(9) = 23.745; *p *= 0.005). SVM shows the highest AUC, however an extreme low value removes the significance of the differences with the AUC distributions from the other classifiers. LDA, LR, MLP, RBF and RF are a homogenous group statistically different from the group composed by QDA, CHART and CHAID. QUEST had the significantly lowest AUC.

### Classification by chance alone

**Figure 8:**
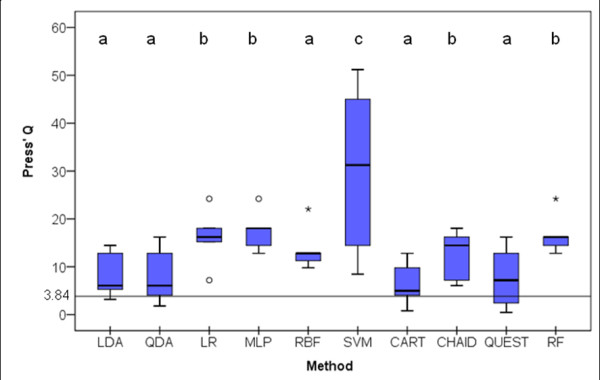
**Box-plot distributions of Press' Q (see text for abbreviations) (X^2^Fr(9) = 21.582; *p *= 0.01)**. Different letters indicate statistically significant differences between classifiers on Dunn's multiple mean rank comparison procedure. Classifiers with Q3.84 classify significantly better than chance alone for a 0.05 significance level. Circles and stars represent outliers and extreme outliers respectively.

Press' Q evaluates the performance of a classifier as compared to chance alone. The test statistic is

$$Q=\frac{{\left(N-nk\right)}^{2}}{N\left(k-1\right)}\sim \chi_{\left(1\right)}^{2}$$

where *N *is the total sample size, *n *is the number of observations correctly classified and *k *is the number of groups. Under the null hypothesis that the classifier is no better than chance alone, Press' Q has a chi-square distribution with 1 degree of freedom. Thus, classifiers with Q≥3.84 classify significantly better than chance alone for a 0.05 significance level. The Q distributions in the 5 sample tests are shown in Figure 8. There were statistically significant differences between the Q distributions (X^2^Fr(9) = 21.582; *p *= 0.01). Dunn's multiple mean rank comparisons revealed that SVM had the highest mean rank followed by RF, MLP, CHAID and LR. The smallest mean ranks were observed for LDA, QDA, RBF, CART and QUEST. All classifiers, with the exception of QUEST, had 1^st ^quartiles higher than 3.84 (*p *< 0.05).

## Discussion

All classifiers evaluated showed better median (Me) classification than chance alone in the prediction of evolution into dementia of elderly people with Mild Cognitive Impairment. Median Press's Q statistic was larger or equal to 5 for all classifiers, although in QUEST the 1^st ^quartile was below the critical level for this statistics. Discriminant power of the classifiers, as judged by the AUC, was appropriate for most classifiers (greater than 0.7) with the exception for classification trees (median AUC of 0.6). No statistically significant differences were found in the total accuracy of 8 of the 10 evaluated classifiers (Medians between 0.63 and 0.73), but RF (Me = 0.74) and SVM (Me = 0.76) obtained statistically significant higher classification accuracy. Median specificity ranged from a minimum of 0.64 (CART and LDA) to a maximum of 1 (SVM). With the exception of LDA, CART and QUEST, all the other classifiers were quite efficient in predicting group membership in the group with larger number of elements (the MCI group corresponding to 69% of the sample) (Median specificity larger than 0.6). Judging from total accuracy, SVM and RF rank highest amongst the classifiers tested as has been suggested elsewhere [47,48,57,58]. However, a quite different picture emerges from the analysis of the sensitivity of the classifiers. Prediction for the group with lower frequency (the Dementia group, 31% of the sample) was quite poor for several of the tested classifiers, including the ones with some of the highest specificity values. Minimum median sensitivity was 0.30 (SVM) and maximum median sensitivity was 0.66 (QUEST, followed by 0.64 for LDA and RF). Only six of the ten classifiers tested showed median sensitivity larger than 0.5 (and only five had 1^st ^quartile sensitivity larger than 0.5). Considering that conversion into dementia is the key prediction in this biomedical application and thus higher sensitivity of classifiers is required, classifiers like Logistic Regression, Neural Networks, Support Vector Machines and CHAID trees are inappropriate for this type of binary classification task. Similar findings were observed in studies comparing different classifiers in other biomedical conditions [24,34,58]. Total accuracy of classifiers is misleading since some classifiers are good only at predicting the larger group membership (high specificity) but quite insufficient at predicting the smaller group membership (low sensitivity). Some of the classifiers with the highest specificity (Neural Networks (MLP and RBF) and SVM) are also the classifiers with the lowest sensitivity. Unbalance of classification efficiency for small frequency vs. large frequency groups has been found in other real-data studies for Logistic Regression and Neural Networks [30,34,59,60]. To our knowledge, such unbalance of SVM in the prediction of the lowest frequency was not been published elsewhere. David Meyer (Personal communication) has observed also that SVM predict poorly low frequency groups. Taking into account total accuracy, specificity and sensitivity, the oldest Fisher's Linear Discriminant Analysis does not rank much lower than Multiple Layer Perceptrons or Random Forests, the newest member of the binary classification family. The relatively small sample size, although in the range of most biomedical experimental studies with dementia and cognitive impairment, may limit the performance of some data mining methods assessed in this study. Sample size has been known to play an important role in the accuracy of Neural Networks [61,62]. In our study, the number of cases for the training and testing sets are at lower limit for recommended data set dimensions for Neural Networks applications (several hundred) [61-63]. Large data sets requirements are also found in LR, but less in LDA if the model assumptions are met. The present sample size was not, apparently, limiting for the achievement of an acceptable accuracy, specificity and sensitivity of both Random Forests and LDA, as reported elsewhere [18,63]. Furthermore, there are studies with relatively small samples where data mining techniques, like SVM and Neural Networks have been used with high accuracy in classification problems [see e.g. [58,64-66]]. Equivalent or even superior performances have been reported for Linear Discriminant Analysis and Random Forests when compared with Neural Networks, Classification Trees and Support Vector Machines [see e.g. [34,47,58,67,68]]. However, controversy still prevails regarding the effects on classifiers' performance of different combinations of predictors, data assumptions, sample sizes and parameters tuning [16,17,31,58,69,70]. Different application with different data sets (both real and simulated) have failed to produce a classifier that ranks best in all applications as shown in the studies by Michie et al., [71] (STALOG project with 23 different classifiers evaluated in 22 real datasets); Lim et al [72] (33 classifiers evaluated on 16 real data sets) and Meyer et al. [34] (24 classifiers, available in the R Software, evaluated on 21 data sets).

It must be pointed out that the results gathered in our study are based on a specific data set and a single set of tuning parameters. It is well known that for Neural Networks and Support Vector Machines the performance of these classifiers and the properties of the resulting predictions are heavily dependent on the chosen values for the tuning parameters [33,34,72,73]. Although, we used settings, that are most commonly used in data mining applications, and tuning parameters, that were optimally determined by grid search methods that minimize total error rates, it may well be that the performance of the data mining methods is just a reflection of the tuning parameters chosen. Discussing Neural Networks versus traditional classifiers, Duin, [73] takes this argument one step further when he states that "(...) a straight forward fair comparison demands automatic classifiers with no user interaction. As this conflicts with one of the main characteristics of neural networks, their flexibility, the question whether they are better or worse than traditional techniques might be undecidable".

Similar results to the ones reported in this study have been made by other authors when classifiers were compared on more than total accuracy or total error rates. For example, Breinman et al. (1984) state that "LDA does as well as other classifiers in most applications". Meyer et al. [34] point out in their comparison study of data mining classifiers, including Neural Networks and SVM, that LDA is a very competitive classifier, producing good results "*out-of-the-box *without the inconvenience of delicate and computationally expensive hyperparameter tuning". In a similar application of Random Forests, SVM, Neural Networks and Linear Discriminant Analysis for recognition of Alzheimer's disease based on electrical brain activity, Lehmann et al. [58] state that "even though modern computer-intensive classification algorithms such as Random Forest, SVM and Neural Networks show a slight superiority, more classical classification algorithms performed nearly equally well".

## Conclusions

For binary classification problems, like prediction of dementia, where classes can be linearly separated and sample size may compromise training and testing of popular data mining and machine learning methods, Random Forests and Linear Discriminant Analysis proved to have high accuracy, sensitivity, specificity and discriminant power. On the contrary, data mining classifiers like Support Vector Machines, Neural Networks and Classification Trees showed low sensitivity, recommending against its use in classification problems where the class of interest is less represented. Since for some data mining techniques the final result and the classifier performance is dependent on the skill of the analyst who applies them and his "special art for tuning the parameters" the question raised by Dunn [33] if "A data mining method can outperform the traditional classifiers?" may well not be ever deniable. However, it is noteworthy to mention that Fisher's Linear Discriminant Analysis, a classifier devised almost a century ago, stands up against computer intensive classifiers, as a simple, efficient, user- and time-proof classifier.

## Competing interests

The authors declare that they have no competing interests. The "Comissão de Ética para a Saúde, Centro Hospitalar Lisboa Norte" ethical committee approved this study.

## Authors' contributions

JM has setup the conceptual research design, did most of the data analysis and interpretation and wrote the first draft of the manuscript; DS collected most of the data, collaborated on data analysis and in the writing and revision of the manuscript; AR collected some of the data; MG collaborated on the conceptual design of the research and in the critical revision of the manuscript for important intellectual content; IS collaborated in the funding of the project and data collection; AdM was responsible for the project design and funding and collaborated in the writing and critical revision of the manuscript for important intellectual content. All authors read and approved the final manuscript.
